# Alpha-Hederin, the Active Saponin of *Nigella sativa*, as an Anticancer Agent Inducing Apoptosis in the SKOV-3 Cell Line

**DOI:** 10.3390/molecules24162958

**Published:** 2019-08-15

**Authors:** Anna Adamska, Justyna Stefanowicz-Hajduk, J. Renata Ochocka

**Affiliations:** Department of Biology and Pharmaceutical Botany, Medical University of Gdańsk, Al. Hallera 107, 80-416 Gdańsk, Poland

**Keywords:** RTCA, MTT assay, flow cytometry, caspases, MMP, cell cycle, annexin, triterpene saponin

## Abstract

Alpha-hederin (α-HN), a pentacyclic triterpene saponin, has recently been identified as one of the active compounds of *Nigella sativa*, as a potential anticancer agent. However, no extensive studies on α-HN have been done as yet, as it was in the case of thymoquinone—the main ingredient of the *N.* *sativa* essential oil. To our knowledge, there are also no data available on how α-HN acts on the human cancer ovarian cell line SKOV-3. In this study we attempt to present the cytotoxic influence of α-HN on the SKOV-3 cell line by means of two methods: Real-Time xCELLigence and 3-(4,5-dimethylthiazol-2-yl)-2,5-diphenyltetrazolium bromide (MTT) assay. The obtained IC_50_ values are 2.62 ± 0.04 μg/mL and 2.48 ± 0.32 μg/mL, respectively. An induction of apoptosis in SKOV-3 cells was confirmed by staining cellular nuclei with Hoechst 33342 dye and by flow cytometry analysis by binding annexin V to the cell membranes. We found that α-HN induces apoptosis in a dose-dependent manner. In the first stages of apoptosis, the mitochondrial membrane potential was found to decrease. Also, inactivation of anti-apoptotic protein Bcl-2 was observed, as well as the caspase-9 and then caspase-3/7 activation. In addition, the treatment of SKOV-3 cells with α-HN induced the cell cycle arrest of cancer cells in G0/G1 phase. The results of our investigations indicate that α-HN induces apoptosis in the SKOV-3 cell line and that the intrinsic mitochondrial pathway is involved in the programmed cancer cell death.

## 1. Introduction

**Figure 1:**
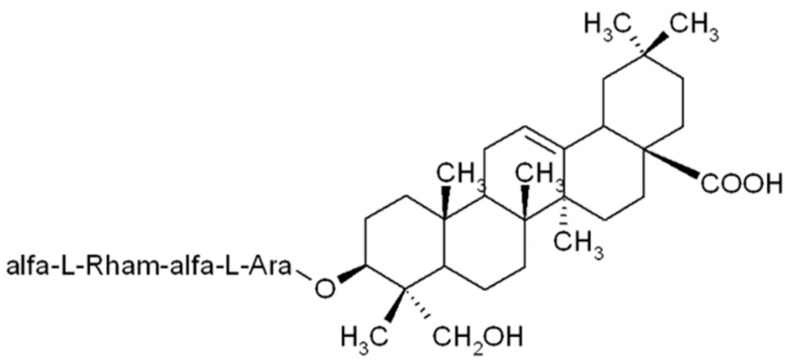
The structure of alpha-hederin (α-HN) [1].

Alpha-hederin (α-HN) (C_41_H_66_O_12_) is found in *Hedera helix*, *Chenopodium quinoa*, *Kalopanax pictus*, and *Nigella sativa*; it is a monodesmosidic triterpenoid saponin. It is also known as (3β,4α)-3-[[2-*O*-(6-deoxy-α-l-mannopyranosyl)-α-l-arabinopyranosyl]oxy]-23-hydroxyolean-12-en-28-oic acid (Figure 1). It appears as a white crystalline powder; its molecular weight is 750.96; its melting point is 128–268 °C; and it is soluble in di-methylformamide, di-methylsulfoxide, and ethanol [1].

α-HN has potential for many significant biological activities. It has recently been identified as another active component, besides thymoquinone, of *N. sativa* seeds, although not much has been disclosed as yet. However, it is now an established fact that saponin possesses anti-inflammatory, anti-arthritic, antioxidant, cytotoxic, and antitumor properties; it can also combat parasites and fungi and is a desmutagenic and hepatoprotective agent [2,3,4,5,6,7,8,9,10,11,12].

As far as an anticancer property of α-HN is concerned, not much research has been done in that regard, and as such, the mechanism of its cytotoxic activity is yet to be established. Lorent et al. claim that α-HN triggers membrane pore formation and it then enters into the cell. presumably through a sterol-dependent pathway [13,14]. It has also been proven that α-HN causes changes in the cell membrane with the cytoplasm vacuolization leading to cell death in cancer cells (melanoma) and non-cancer (mouse 3T3 fibroblasts) cells [11]. Jeong HG and Choi CY stated that α-HN can upregulate the expression of iNOS (inducible nitric oxide [NO] synthase) through NF-*κ*B transactivation and thereby stimulate the release of NO, which contributes to the oxidative damages of cells in mouse macrophages [15]. Also, α-HN together with pentoxifylline has been proven to be cytotoxic, as it downregulates the IL-6 and TNF-α mRNA levels in murine macrophage (RAW 264.7) and murine hepatoma (Hepa-1c1c7) cell lines [9]. Besides, in the α-HN’s mechanism of cytotoxic action on murine leukemia P388 cells [16], the generation of reactive oxygen species and depletion of intracellular glutathione also play a role. It is also evident that α-HN activates caspase-3 and caspase-9 via the depolarization of the mitochondrial membrane potential, which triggers the release of the apoptosome (Apaf-1) and cytochrome c in human breast cancer cells (MDA-MB-231 and MCF-7) [17]. In larynx carcinoma (HEp-2), α-HN elicits apoptosis and necrosis [12]. α-HN is also active against other cell lines, for instance: Lewis lung carcinoma (LL/2), smooth muscle (HASM), human kidney (HEK293), and transfected HEK293 cell lines [18,19]. With regard to colon cancer (HT-29), α-HN is claimed to act synergistically with 5-fluorouracil and 3-*O*-α-l-rhamnopyranosyl-(1→2)-α-l-arabinopyranoside [20,21]. Furthermore, α-HN was also tested in comparison to its chitosan nanoparticles (α-HN-CS-CD147-NPs) on human liver cancer cell lines SMMC-7721 and HepG2 and it was found that the antibody when coupled with α-HN became more active against these cell lines than α-HN alone [22]. 

Not only does α-HN possess the potential for numerous biological activities in vitro, but also it is an efficacious agent in many studies in vivo; for instance, in the treatment of asthma in a rat ovalbumin (OVA)-sensitized model of asthma. The results have shown that in a group of rats pretreated with α-HN the IL-17 mRNA levels were decreased and miRNA-133a gene expression was increased in comparison to the OVA-sensitized group of rats [23].

Despite a few studies available on α-HN as an anti-cancer agent on various cell lines, no data can be found about its impact on human ovarian cancer cell line SKOV-3. SKOV-3 (or interchangeably SK-OV-3) lines were first derived from ovarian cancer cells in 1973 from a 64-year-old woman who showed high resistance to numerous cytotoxic agents, including TNF, adriamycin, and cisplatin [24,25]. Additionally, the SKOV-3 cell line is suitable for preclinical testing, characterized by similarity to ovarian cancer in vivo and shows high ability to form tumors in mice after injection at specific sites [26].

This study investigates the cytotoxic effect of α-HN on the SKOV-3 cell line and examines the type of cell death and mechanism of its action on the cells.

## 2. Results

### 2.1. α-HN Decreased SKOV-3 Cells’ Viability

The effect of α-HN, the active triterpenoid saponin of *N. sativa* seeds, on the viability of the cells, was tested by the Real-Time xCELLigence system that works based on electronic impedance that is measured in E-plate wells equipped with sensor electrodes. It allows monitoring the cells continuously and quantitatively [27]. Any alteration in cells’ viability, morphology, number, or degree of adhesion has an impact on the electrode’s impedance, which is represented by the parameter CI [28,29]. The IC_50_ value is calculated on the basis of CI at every measuring point of the experiment.

**Figure 2:**
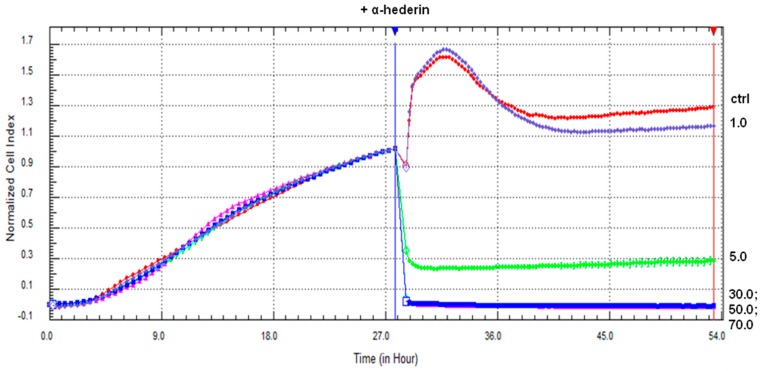
The SKOV-3 cells’ viability declines after the treatment with α-HN. The SKOV-3 cells were incubated with α-HN for 24 h. The curves are labeled with numbers that represent increasing concentrations of α-HN (1–70 µg/mL, respectively).

**Figure 3:**
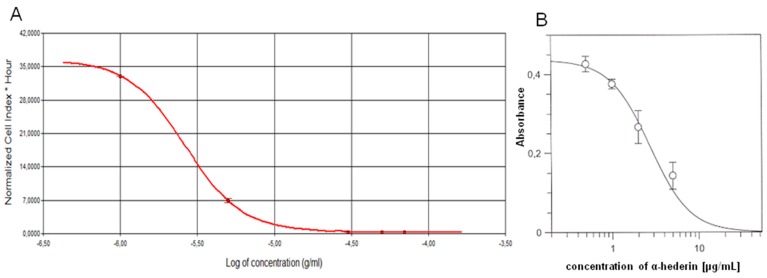
The dose-response curves obtained from the RTCA system and MTT assay after the SKOV-3 cells’ treatment with α-HN. α-HN was used in concentrations of 1–70 and 0.5–50 µg/mL, respectively. IC_50_ values of α-HN were measured on the grounds of the dose-response curves by the Real-Time xCELLigence system (**A**) and MTT assay (**B**). Standard deviations are represented by error bars.

SKOV-3 cells were treated with α-HN (1–70 µg/mL) for 24 h (Figure 2). The obtained IC_50_ value of α-HN by using the RTCA system was 2.62 ± 0.04 µg/mL, based on the sigmoidal dose-response formula (Table 1, Figure 3).

The MTT assay was performed with α-HN at the concentration range 0.5–50 µg/mL, for 24 h, to confirm the RTCA results of the cytotoxic activity of α-HN on SKOV-3 cells. The obtained IC_50_ value was 2.48 ± 0.32 µg/mL (Table 1, Figure 3). A control sample (DMSO 0.5% (*v*/*v*)) did not inhibit the cell growth.

Additionally, we prepared the same experiments using the non-tumor HaCaT cell line. The results collected from RTCA and MTT assays were 2.71 ± 0.35 and 2.57 ± 0.21 µg/mL, respectively (Table 1).

The obtained results indicate that SKOV-3 and HaCaT cells were sensitive to α-HN and the viability was significantly decreased in both the lines after treatment with the compound. This effect was time and dose-dependent.

### 2.2. α-HN Triggered Changes in SKOV-3 Nuclei

**Figure 4:**
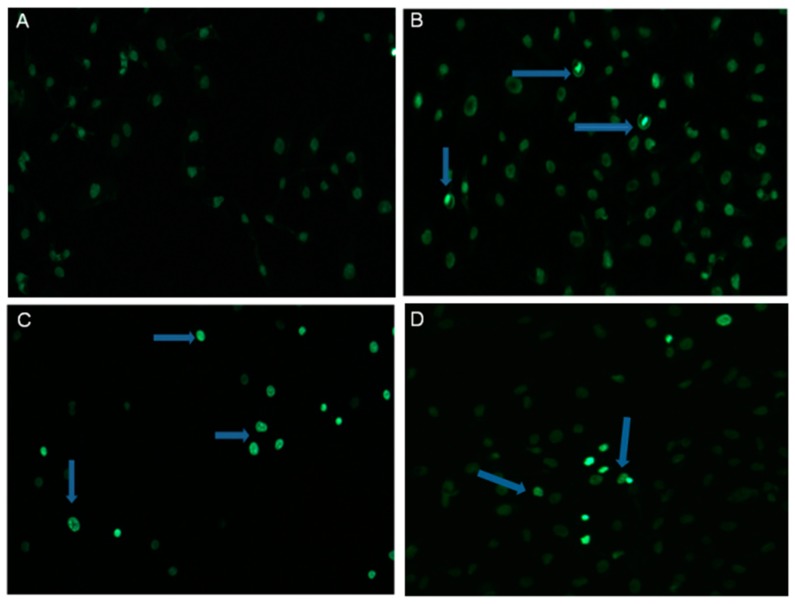
Apoptotic changes induced in SKOV-3 cells by α-HN. The state of SKOV-3 nuclear chromatin after treating with 0.1% DMSO (**A**) or α-HN at concentration of 0.5, 2 and 10 µg/mL (**B**–**D**) was evaluated by Hoechst 33342 staining. The cells after treatment with α-HN depict condensed chromatin (contrary to the DMSO control cells). Arrows point out apoptotic cells.

The changes in chromatin distribution in SKOV-3 cells after treating with α-HN were evaluated by staining the cellular nuclei with Hoechst 33342 dye. The nuclear fragmentation and chromatin condensation were visible in SKOV-3 cells after staining, contrary to the control sample with DMSO (Figure 4).

### 2.3. α-HN Induced Apoptosis in the SKOV-3 Cell Line

**Figure 5:**
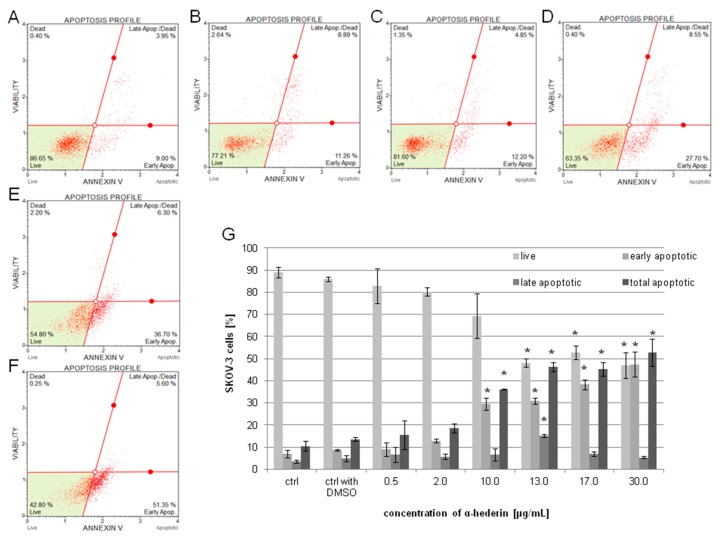
Apoptosis assay on SKOV-3 cells treated with α-HN. The SKOV-3 cells were examined by flow cytometry using annexin V/7-AAD staining method. The percentage of apoptotic cells was estimated in the control sample of the untreated cells (control), after the cells’ incubation with DMSO (control with DMSO) at concentration of 0.5% (**A**) or with α-HN at concentrations of 0.5–30 μg/mL for 24 h (particular concentrations are represented in pictures: 0.5 (**B**), 2 (**C**), 10 (**D**), 17 (**E**), and 30 μg/mL (**F**)). The mean values of three independent trials of the total apoptotic rate were calculated in comparison to the DMSO control (**G**). Standard deviation is represented by error bars. Significant differences relative to the DMSO control are marked with an * (*p* < 0.05).

The induction of apoptosis in the SKOV-3 cells was confirmed by a staining test (after cells’ incubation with 0.5–30 µg/mL of α-HN for 24 h) with annexin V/7-AAD and then examined by Muse Cell Aanalyzer. An increase in a dose-dependent manner in the percentage of apoptotic cells was observed. The total percentages, as mean values of three independent experiments, of apoptotic cells (early and late apoptotic) were 15.55% ± 6.51%, 18.50% ± 2.04%, 36.1% ± 0.21%, 46.13% ± 2.09%, 45.23% ± 3.15%, and 52.63% ± 6.12% with α-HN concentrations of 0.5, 2, 10, 13, 17, and 30 µg/mL, respectively, in comparison to the untreated SKOV-3 cells and the cells treated with DMSO: 10.42% ± 2.26%, and 13.6% ± 0.92%, respectively (Figure 5).

### 2.4. α-HN Decreased Mitochondrial Membrane Potential (ΔΨm) in SKOV-3 Cells

**Figure 6:**
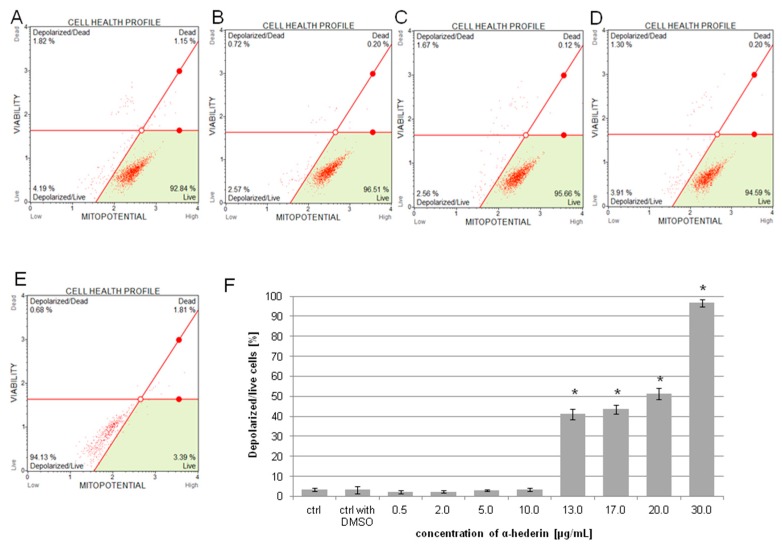
α-HN triggered alterations in the inner transmembrane mitochondrial potential in SKOV-3 cells. The loss of potential was determined after 3 h in untreated SKOV-3 cells (control), in the cells treated with 0.5% DMSO (control with DMSO) (**A**), and within the cells treated with increasing concentrations of α-HN in the range 0.5–30 μg/mL (particular concentrations are represented in pictures: 0.5 (**B**), 2 (**C**), 10 (**D**), and 30 μg/mL (**E**)). The measurement of SKOV-3 mitochondrial depolarization was evaluated in relation to the 0.3% DMSO control (**F**). Each sample was run three times, independently. Standard deviation is represented by error bars. Significant differences relative to the control are marked with an * (*p* < 0.05).

Mitochondrial loss of inner transmembrane potential, which demonstrates mitochondrial dysfunction, is one of the hallmarks of the early stages of apoptosis. The condition of mitochondrial membranes of SKOV-3 cells after treatment with α-HN was evaluated by flow cytometry. The inner membranes of intact mitochondria accumulated fluorescent dye, which was visible in the control samples. The cells incubated with the increasing concentrations of α-HN depicted declining fluorescence. The mean values of depolarized/live cells after treatment with α-HN were 2.24% ± 0.73%, 2.35% ± 0.74%, 3.11% ± 0.40%, 3.37% ± 0.93%, 41.15% ± 2.57%, 43.51% ± 2.25%, 51.25% ± 2.84%, and 96.79% ± 1.83% for concentrations of 0.5, 2, 5, 10, 13, 17, 20, and 30 μg/mL, respectively (Figure 6). The acquired data confirm the participation of mitochondria in the apoptosis induction in SKOV-3 cells after treatment with α-HN.

### 2.5. α-HN Inactivated Bcl-2 Protein

The total level of Bcl-2, an anti-apoptotic family member, expression was measured according to the Muse Activation Dual Detection Kit, which utilizes a pair of antibodies: One antibody enables the detection of total protein expression and the other allows the detection of the phosphorylated form (activated) of the same target, which gives an overview of whole Bcl-2 activation pathway measured simultaneously in one cell.

**Figure 7:**
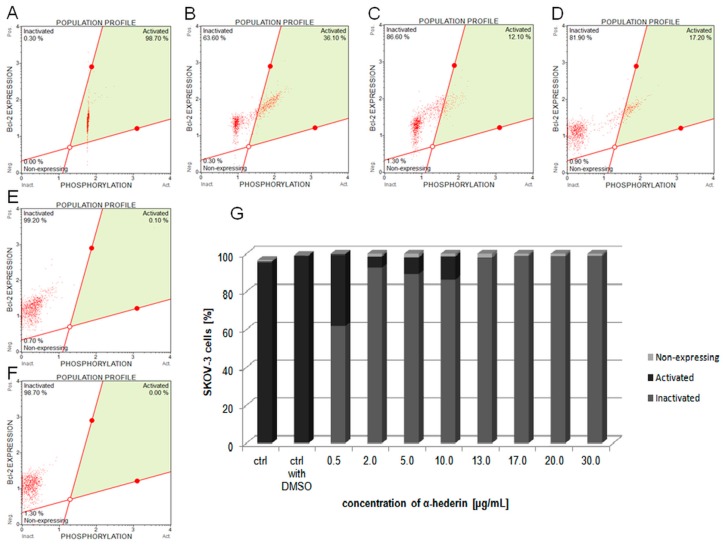
Estimation of Bcl-2 inactivation in SKOV-3 cells treated with α-HN. The diagrams depict the mean values of three independent experiments with the levels of anti-apoptotic protein, Bcl-2, which was measured in SKOV-3 cells that were previously treated with 0.3% DMSO (control with DMSO) (**A**) and the increasing concentrations of α-HN in the range 0.5–30 µg/mL for 24 h (particular concentrations are represented in pictures: 0.5 (**B**), 2 (**C**), 10 (**D**), 17 (**E**), and 30 μg/mL (**F**)). The results are presented as percentages of non-expressing, activated (via phosphorylation), and inactivated cells (**G**). Control: The untreated cells.

SKOV-3 cells were exposed to the increasing concentrations of α-HN (0.5–30 µg/mL) for 24 h to trigger cell death and to inhibit the response of the Bcl-2 signaling cascade. The flow cytometry data depict the relative values (in percentage) for each population of cells: Activated, inactivated, and non-expressing Bcl-2. In our study, a significant dose-dependent inactivation of the anti-apoptotic protein was observed, contrary to the control samples with almost 100% of Bcl-2 activation rate. The percentages of Bcl-2 inactivation measured were 61.87% ± 3.35%, 92.65% ± 8.56%, 89.3% ± 11.88%, 86.37% ± 10.26%, 98.03% ± 0.7%, 99.07% ± 0.51%, 98.8% ± 0.84%, and 99.07% ± 0.4% with α-HN concentrations of 0.5, 2, 5, 10, 13, 17, 20, and 30.0 µg/mL, respectively (Figure 7).

### 2.6. α-HN Induced Changes in the Caspases’ Activity

**Figure 8:**
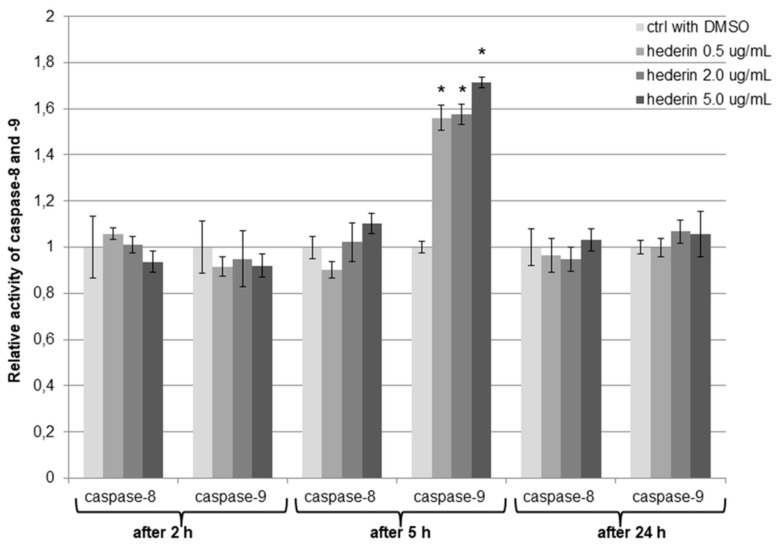
Caspase-8 and caspase-9 activity after the SKOV-3 cells’ treatment with α-HN. A significant increase was observed in caspase-9 activity in the SKOV-3 cell line after 5 h of incubation with different concentrations of α-HN in comparison to the control (0.3% DMSO-treated cells). Each sample was run three times, independently. Error bars represent standard deviations. Significant differences relative to the DMSO control are marked with * (*p* < 0.05).

In order to estimate the activity of caspase-8 and caspase-9, the SKOV-3 cells were treated with 0.5–30 µg/mL of α-HN for 2, 5, and 24 h. In the case of caspase-8 in comparison to the control (DMSO-treated cells), no significant activation was observed. The activity of caspase-8 for the concentrations 0.5, 2, and 5 µg/mL of α-HN was similar to the control (Figure 8); however, for the higher concentrations of α-HN (in the range 10–30 µg/mL) and at all the time measurement points, the activity was near value 0 (data not shown).

As far as caspase-9 is concerned, there was a significant increase in its activation after 5 h of SKOV-3 cells’ treatment with α-HN for the concentrations of 0.5, 2, and 5 µg/mL. The activity of caspase-9 was 1.56, 1.58, and 1.71-fold higher, respectively, in comparison to the DMSO control (Figure 8). Similar to the caspase-8 luminometric results, the activity of caspase-9 was near value 0 for the higher concentrations of α-HN (in the range 10–30 µg/mL) for each measuring point (data not shown).

**Figure 9:**
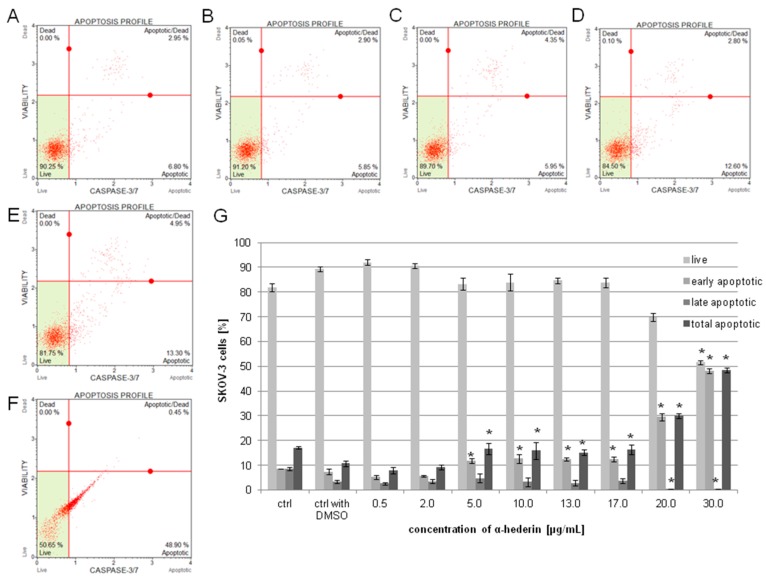
Caspase-3/7 activation in SKOV-3 cells treated with α-HN. Diagrams depict the mean values of early, late, and totally apoptotic cells of three independent experiments after incubation of the SKOV-3 cells with 0.3% DMSO (control with DMSO) (**A**) and increasing concentrations of α-HN in the range 0.5–30 µg/mL for 24 h (particular concentrations are represented in pictures: 0.5 (**B**), 2 (**C**), 10 (**D**), 17 (**E**), and 30 μg/mL (**F**)). Significant differences relative to DMSO control are marked with * (*p* < 0.05) (**G**). Error bars represent standard deviation. Control: the untreated cells.

In order to estimate the activity of caspase-3/7, SKOV-3 cells were treated with increasing concentrations of α-HN for 24 h and then stained with the Muse Caspase-3/7 Kit and analyzed with the Muse Cell Analyzer. The percentages of the cells in different stages of apoptosis were determined based on the activation of caspase-3/7. Four types of cell populations were registered: Live, early apoptotic, late apoptotic, and dead. The total percentages, as mean values of three independent experiments, of apoptotic cells (early and late apoptotic) were 7.93% ± 1.20%, 9.3% ± 0.92%, 16.7% ± 2.41%, 16.0% ± 3.44%, 15.23% ± 1.14%, 16.27% ± 1.93%, 30.12% ± 0.95%, and 48.42% ± 0.95% with α-HN concentrations of 0.5, 2, 5, 10, 13, 17, 20, 30 µg/mL, respectively, in comparison to the untreated SKOV-3 cells and the cells treated with DMSO: 17.13% ± 0.46% and 10.72% ± 1.03%, respectively (Figure 9).

### 2.7. Arresting the Cell Cycle

The data acquired from cell cycle analysis by the Muse Cell Analyzer depict a crucial dose-dependent increase of SKOV-3 cells in the G0/G1 population, as a result of arresting the cell cycle in this phase after 24 h exposure to the increasing concentrations of α-HN.

**Figure 10:**
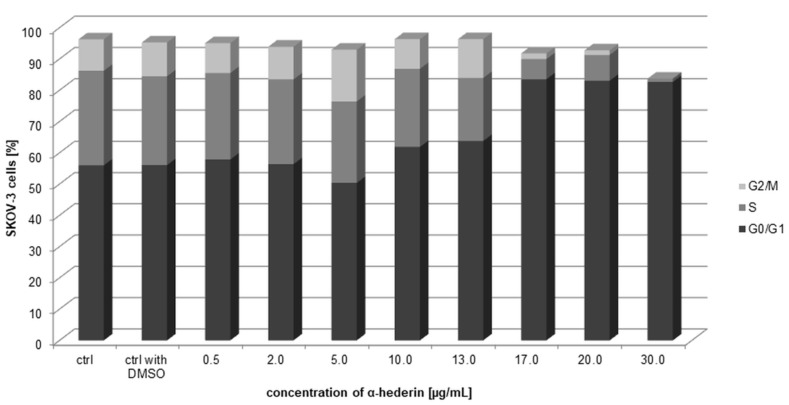
Cell cycle analysis of the SKOV-3 cells treated for 24 h with increasing concentrations of α-HN. A dose-dependent increase of the cells in G0/G1 populations was compared with that of the untreated cells (control) and the DMSO control sample (control with DMSO). Diagram depicts the mean values of percentages of three independent analysis of the cells accumulated in particular cell cycle phase.

After 24 h treatment, SKOV-3 cells indicated percentages (the mean value of three independent experiments) of G0/G1 populations: 57.83% ± 4.75%, 56.35% ± 5.94%, 50.35% ± 0.07%, 61.86% ± 8.62%, 63.75% ± 0.35%, 83.5% ± 2.26%, 83% ± 1.13%, 82.65% ± 2.19% for the concentrations of α-HN: 0.5, 2, 5, 10, 13, 17, 20, 30 µg/mL, respectively; compared to the control of untreated cells 55.98% ± 2.24% and the cells treated with DMSO 56.05% ± 2.17% (Figure 10). A significant accumulation of the cells in G0/G1 phase can be seen, specially beginning with the dose of 10 µg/mL of α-HN, and most crucially in a range 17–30 µg/mL.

## 3. Discussion

*Nigella sativa* seeds have been used since ancient times to cure many diseases, as there were believed to have stimulatory, choleretic, cholagogue, carminative, diuretic, and diaphoretic properties. In folk medicine, seeds were meant to be the treatment for and prevention option for dyslipidemia, asthma, and eczema [30]. The seeds and the main component of the volatile oil, thymoquinone, were also widely explored in numerous scientific studies, also for its cytotoxic characteristics [31,32,33,34,35,36,37,38].

However, limited attention has been paid to the active saponin of *N. sativa* seeds, α-HN (a triterpenoid with hederagenin aglycone), especially as far as anticancer properties are concerned and the mechanisms of that activity. Apart from its scientifically researched anti-inflammatory, antioxidant, antiarthritic, antiparasitic, antifungal, bronchiolytic, and hepatoprotective potential [1], it was proven that α-HN inhibits the proliferation of particular cell lines [18,19].

To our knowledge, not much information is available about the influence of α-HN on the SKOV-3 ovarian cell line [20] and also no data are available about the mechanism underlying this effect.

The SKOV-3 cell line was tested for cytotoxicity with triterpenoid saponins, but not those with hederagenin aglycone. The lupane and oleane-type saponins were isolated from *Pulsatilla koreana* roots [39,40] and tested on, among others, the SKOV-3 cell line. On the other hand, saponins with hederagenin aglycone were also examined for its cytotoxicity, though not on the SKOV-3 cell line, and α-HN proved to be the most potent among other related hederagenin diglycosides [41].

In this study, we examined the impact of α-HN on the ovarian cell line, SKOV-3, and estimated the mechanism of its anticancer activity. Our results show that this triterpenoid saponin exhibits crucial cytotoxic activity against SKOV-3 cells. The proliferation of the tested cells assessed through the Real-Time xCELLigence system and MTT assays was strongly inhibited in a dose and time-dependent manner.

Our investigations also indicate that the type of cell death induced by α-HN in SKOV-3 cells is apoptosis. This programmed cell death is distinguished by specific biochemical and morphological hallmarks, such as cell shrinkage, formation of condensed nuclei followed by nuclear fragmentation and membrane blebbing [42]. In our study, all these nuclear changes were observed by staining SKOV-3 cells with the fluorescent dye after incubating the cells with α-HN.

Additionally, an early apoptosis hallmark is the loss of plasma membrane asymmetry with the cell membrane phosphatidylserine translocation from the inner to the outer surface. As a result, a dependent phospholipid-binding protein, called annexin V, can bind to the phosphatidylserine [43,44,45]. Our data obtained from the experiment depict a crucial increase in the population of apoptotic SKOV-3 cells after treatment with α-HN in a dose-dependent way.

As far as the main pathways of apoptosis are concerned, there are the extrinsic, via the death receptor pathway, and the intrinsic, via the mitochondrial pathway [46,47,48]. In terms of the extrinsic pathway, the ligand is attached to the membrane death receptor, which triggers changes in the receptor’s death domain, hence the caspase-8 is activated as well. This gives a signal to initiate apoptosis. As for the intrinsic mitochondrial-dependent pathway, as a result of the chemical stimulus, numerous proteins are released from the mitochondria into the cytoplasm. Hence, a loss of the mitochondrial inner transmembrane potential (ΔΨm) can be observed [49], which is also associated with the apoptosis early stages [50,51,52]. This process is closely related to the role of both anti and pro-apoptotic Bcl-2 proteins that are located in the outer mitochondrial membrane and/or in the cytosol.

The ability of the Bcl-2 protein to inhibit the apoptosis is regulated by the phosphorylation process. Its phosphorylated form has an anti-apoptotic function and dephosphorylation is required for pro-apoptotic activity [53,54]. This process leads to the formation of pores in the mitochondrial membrane and to the release of cytochrome c from mitochondria to the cytosol. Released cytochrome c, together with the apoptotic protease activation factor Apaf-1 protein, and ATP, creates a structure called the apoptosome. In the next stage, oligomerization of the Apaf-1 protein takes place, and a number of procaspase-9 molecules are attached. The procaspase-9 molecules undergo self-activation, and this new structure is catalytically active for a longer time. The primary role of apoptosome is the activation of executive caspases, including caspase-3—the essential executive enzyme of the process of apoptosis [55,56,57,58,59,60,61,62,63].

In our study, a crucial dose-dependent depolarization of the mitochondrial membrane in the SKOV-3 cell line was detected in a few hours after treating the cells with the saponin, together with inactivation of the anti-apoptotic Bcl-2 protein. Moreover, a significant increase in the caspase-9 activity was observed and this led to caspase-3/7 activation in the cells, which confirms the initiation of the intrinsic mitochondrial pathway in SKOV-3 cell line death. Simultaneously, no activation of caspase-8, involved mainly in the extrinsic apoptosis pathway, was observed.

In the research performed by Sun J. et al., α-HN also showed strong anticancer-effect on colorectal cancer cells (HCT116 and HCT8) and induced apoptosis via ROS-activated (reactive oxygen species) mitochondrial signaling pathway and autophagic cell death through ROS dependent AMPK/mTOR signaling pathway activation [64]. Moreover, similar to our results, Wang H. et al. proved that α-HN significantly enhanced caspase-3 and -9 activity, whereas the Bcl-2 level was lowered in oral cancer SCC-25 cells. The authors also showed that the apoptosis in these cells was regulated by the PI3K/Akt/mTOR signaling pathway [65].

In this study, the cell cycle arrest in SKOV-3 cells was also observed in G0/G1 phase after treatment for 24 h with increasing concentrations of α-HN. In another study conducted by Sun D. et al., α-HN inhibited the cell cycle in G2/M phase and induced apoptosis in colon cancer cells (SW620) via mitochondrial and caspase-dependent pathway together with disruption of NF-*κ*B and ERK pathways [66].

To sum up, our investigations proved that α-HN induces apoptosis in the SKOV-3 cell line and that the intrinsic mitochondrial pathway plays a significant role in this process. However, further investigation into the programmed death of ovarian cancer cell line SKOV-3 is required, since no more related data can be found.

## 4. Materials and Methods

### 4.1. Materials

McCoy’s 5A Medium DMEM (Dulbecco’s Modified Eagle’s Medium), streptomycin, penicillin, PBS (phosphate-buffered saline), DMSO (dimethyl sulfoxide), MTT (3-(4,5-dimethylthiazol-2-yl)-2,5-diphenyltetrazolium bromide) and α-HN were purchased from Sigma-Aldrich (St. Louis, MO, USA). α-HN was dissolved in DMSO (99.9%) to the final concentration of 10 mg/mL.

### 4.2. Cell Line Culture

The ovarian cancer cell line (SKOV-3) and the human keratinocytes (HaCaT) were obtained from the American Type Culture Collection (ATCC, Manassas, VA, USA).

The SKOV-3 cells were cultured in McCoy’s medium supplemented with 10% (*v*/*v*) FBS (fetal bovine serum), 100 units/mL of penicillin, and 100 µg/mL of streptomycin. The human keratinocytes (HaCaT) were cultured in DMEM medium supplemented with 10% (*v*/*v*) FBS, 100 units/mL of penicillin, 100 µg/mL of streptomycin and 2 mM L-glutamine. All the cultures were kept in a humidified 5% CO_2_ incubator at 37 °C.

### 4.3. Cell Viability Assays

#### 4.3.1. Real-Time xCELLigence Cell Proliferation Assay

The Real-Time xCELLigence system (ACEA Biosciences, San Diego, CA, USA) is devoted to monitoring cytotoxicity and is based on electronic sensor array technology. All the cells were seeded into E-plate 16 (each well with 100 µL medium) at a density of 2 × 10^4^ cells/well. When the cells entered into the log phase, α-HN was added in a range 1–70 µg/mL in at least two independent experiments in duplicate (*n* = 4). DMSO concentration in the control did not exceed 0.7% (*v*/*v*). The cells were then incubated with α-HN and monitored for 24 h at 37 °C in a 5% CO_2_ atmosphere. 

The cell index (CI) and IC_50_ values were taken from the measured cell-electrode impedance that is directly related to the viability and number of cells.

#### 4.3.2. MTT Assay

The viability of the cells was also determined by means of MTT assay. The cells were seeded in 96-well plates at a density of 2 × 10^3^ cells/well and then treated for 24 h with α-HN in the concentration range 0.5–50 µg/mL. DMSO was added to the control cells at a concentration of 0.5% (*v*/*v*). Next, the medium with α-HN was discarded and a fresh medium with MTT solution (0.5 mg/mL) was added to the cells and incubated for 3 h at 37 °C. Formazan solution’s optical density was measured at 570 nm by Epoch (BioTek Instruments, Winooski, VT, USA), a plate reader. The results of the three independent experiments (each in six repetitions, *n* = 18) are expressed as IC_50_ mean values (±SD).

### 4.4. Hoechst Staining

The blue fluorescent Hoechst 33342 dye (Life Technologies, Carlsbad, CA, USA) was used to analyze the effect of α-HN on nuclei in SKOV-3 cells. The cells were seeded at a density of 5 × 10^5^ cells/well in 6-well plates and then treated with α-HN dissolved in DMSO at a concentration of 0.5, 2, and 10 µg/mL. DMSO concentration did not exceed 0.1% (*v*/*v*). Then, 24 h later, SKOV-3 cells were stained with 0.5 µg/mL of the Hoechst dye (dissolved in PBS) for 25 min in a CO_2_ incubator and observed under a fluorescent microscope (Leica, Heerbrugg, Switzerland).

### 4.5. Apoptosis Assay

A test involving the binding of annexin V to cellular phosphatidylserine was performed to assess the induction of apoptosis. The SKOV-3 cells with a density of 1 × 10^5^ cells/well were treated with α-HN at a concentration of 0.5–30 µg/mL for 24 h. The DMSO concentration (control sample) did not exceed 0.3% (*v*/*v*). SKOV-3 cells were first harvested, then stained following the Muse Annexin V and Dead Cell Assay Kit (Merck Millipore, Darmstadt, Germany), and analyzed using Muse Cell Analyzer (Merck Millipore). The tests were repeated at least three times independently.

### 4.6. Assessment of Mitochondrial Membrane Potential

The SKOV-3 cells were seeded at a density of 1 × 10^5^ cells/well and then treated with α-HN at a concentration of 0.5–30 µg/mL for 3 h. Such a period of incubation time was selected in order to depict the early stage of mitochondrial dysfunction of the SKOV-3 cells. The DMSO concentration (control sample) did not exceed 0.3% (*v*/*v*). The cells were harvested after 3 h of exposure and prepared, following the manufacturer’s protocol of Muse Mitopotential Kit (Merck Millipore). The percentage of depolarized/live cells was determined using Muse Cell Analyzer. The trials were repeated at least three times independently.

### 4.7. Bcl-2 Activation Dual Detection

The SKOV-3 cells were seeded at a density of 5 × 10^5^ cells/well and then treated with α-HN at a concentration of 0.5–30 µg/mL for 24 h. DMSO (control sample) concentration did not exceed 0.3% (*v*/*v*). The cells were then collected, washed with PBS, and, following the manufacturer’s protocol, the cocktail of specific antibodies was added (a phospho-specific anti-phospho-Bcl-2 (Ser70)-Alexa Fluor 555 and an anti-Bcl-2-PECy5 conjugated antibodies) from the Muse Activation Dual Detection Kit (Merck Millipore). The cells were incubated at room temperature for 30 min in the dark and then the total level of Bcl-2 expression and activated form of this protein was measured using Muse Cell Analyzer. The experiments were repeated minimum three times independently.

### 4.8. Assessment of Caspase-3/7/8/9 Activity

The activity of caspase-8 and caspase-9 was measured after 2, 5, and 24 h of exposure of cells (1 × 10^4^ cells/well) to α-HN at concentration values of 0.5, 2, 5, 10, 13, 17, 20 and 30 µg/mL. The final concentration of DMSO did not exceed 0.3% (*v*/*v*). The cells were then treated with Caspase-Glo 8 or 9 Assay Kits (Promega, Madison, WI, USA) according to the manufacturer’s protocol. The activity of the caspases was analyzed by Glomax Multi+ Detection System (Promega). The experiment was repeated three times, independently.

The caspase-3 and caspase-7 activation was determined by Muse Caspase-3/7 Kit (Merck Millipore). The kit measures two significant parameters of cell health—apoptotic status based on caspase-3/7 activation and cell membrane integrity or cell death. The method allows determining simultaneously the percentage of live, total apoptotic (early and late apoptotic), and dead cells. The cells were seeded at a density of 1 × 10^5^ cells/well in 6-well plates and then treated with α-HN dissolved in DMSO at concentration values of 0.5, 2, 5, 10, 13, 17, 20 and 30 µg/mL. DMSO concentration did not exceed 0.3% (*v*/*v*). After 24 h cells were harvested, stained with Muse reagent, and incubated for 30 min in a CO_2_ incubator; thereafter they were stained with 7-AAD and incubated at room temperature in the dark for 5 min. In the end, SKOV-3 cells were analyzed by Muse Cell Analyzer. The experiment was repeated three times independently.

### 4.9. Arresting the Cell Cycle

Propidium iodide (PI), the nuclear DNA intercalating stain, was used to discriminate SKOV-3 cells at various stages of the cell cycle, following the manufacturer’s protocol of the Muse Cell Cycle Assay Kit (Merck Millipore). The cells were seeded at a density of 5 × 10^5^ cells/well in 6-well plates and then treated for 24 h with α-HN dissolved in DMSO at concentration values of 0.5, 2, 10, 20 and 30 µg/mL. DMSO concentration did not exceed 0.3% (*v*/*v*). SKOV-3 cells were harvested, stained with Muse Cell Cycle reagent following the manufacturer’s protocol, incubated for 30 min at room temperature, and analyzed by Muse Cell Analyzer. The experiments were repeated three times independently.

## Figures and Tables

**Table 1:** The mean values of IC_50_ (µg/mL) of α-HN on the grounds of Real-Time xCELLigence system and MTT assays for SKOV-3 and HaCaT cell lines.

Cell Line	SKOV-3		HaCaT	
**Method**	**RTCA**	**MTT**	**RTCA**	**MTT**
	2.62 * ± 0.04; 1 ^a^	2.48 ** ± 0.32	2.71 * ± 0.35; 0.99 ^a^	2.57 ** ± 0.21

^a^ R^2^: The coefficient of determination. * The average values of IC_50_ from two independent experiments, each run in duplicate. ** The average values of IC_50_ from three independent experiments, each in six repetitions.
